# Basaloid squamous cell carcinoma in the nasal cavity treated with proton beam therapy concurrent with cisplatin: a case report

**DOI:** 10.1186/1752-1947-8-123

**Published:** 2014-04-09

**Authors:** Shigeyuki Takamatsu, Kazutaka Yamamoto, Tamaki Kondou, Mariko Kawamura, Satoko Asahi, Yuuji Tameshige, Yoshikazu Maeda, Makoto Sasaki, Hiroyasu Tamamura, Akira Tsuji, Yasuharu Kaizaki, Tomoyasu Kumano, Tsuyoshi Takanaka

**Affiliations:** 1 Proton Therapy Center, Fukui Prefectural Hospital, 2-8-1 Yotsui, Fukui City, Fukui 910-8526, Japan; 2 Department of Diagnostic and Therapeutic Radiology, Kanazawa Medical University, 1-1 Daigaku, Uchinada, Kahoku, Ishikawa 920-0923, Japan; 3 Division of Otolaryngology Head and Neck Surgery, Graduate School of Medicine, Kanazawa University, 13-1 Takaramachi, Kanazawa city, Ishikawa 920-8640, Japan; 4 Department of Pathology, Fukui Prefectural Hospital, 2-8-1 Yotsui, Fukui City, Fukui 910-8526, Japan; 5 Department of Radiotherapy, Graduate School of Medicine, Kanazawa University, 13-1 Takaramachi, Kanazawa city, Ishikawa 920-8640, Japan

**Keywords:** Basaloid squamous cell carcinoma, Cisplatin, Concurrent radiochemotherapy, Nasal cavity, Proton beam therapy

## Abstract

**Introduction:**

Basaloid squamous cell carcinoma is a rare and aggressive variant of squamous cell carcinoma. Basaloid squamous cell carcinoma is mostly seen in the upper aerodigestive tract and has a propensity for lymph node spread and systemic metastases. Various treatment modalities have been reported, including surgical excision supplemented with radiotherapy/adjuvant chemotherapy. To the best of our knowledge, treatment of nasal basaloid squamous cell carcinoma with proton beam therapy and cisplatin has not been described in the literature.

**Case presentation:**

We report the case of a 56-year-old Japanese man with locally invasive basaloid squamous cell carcinoma in his right nasal cavity with invasion of the orbit, paranasal sinus, and buccal subcutaneous tissue. He underwent proton beam therapy concurrent with cisplatin. Acute and late side effects did not exceed grade 3. At 24-month follow up, he remains in complete remission.

**Conclusion:**

Proton beam therapy concurrent with cisplatin may be one choice for locally invasive basaloid squamous cell carcinoma.

## Introduction

The histological characteristics of basaloid squamous cell carcinoma (BSCC) were first described by Wain *et al.* in 1986 [1]. BSCC is a rare and aggressive variant of squamous cell carcinoma (SCC). BSCC is mostly seen in the upper aerodigestive tract, including the oral cavity, larynx, and hypopharynx [2-4]. BSCC has a propensity for nodal spread and systemic metastases. Various treatment modalities have been reported, including surgical excision supplemented with radiotherapy/adjuvant chemotherapy [2-7]. There is no established consensus on treatment because of the limited number of cases of BSCC. We report the case of a patient with locally invasive BSCC of the nasal cavity who was treated with proton beam therapy (PBT) concurrent with cisplatin (CDDP).

## Case presentation

**Figure 1:**
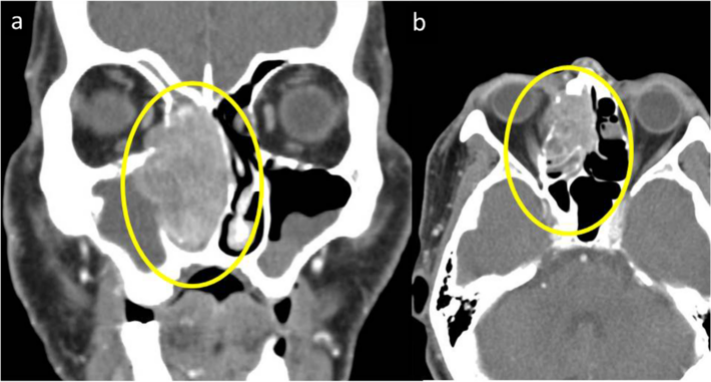
**Contrast-enhanced computed tomography scans. (a)** Contrast-enhanced computed tomography reveals a right nasal cavity tumor with invasion of the right maxillary sinus and frontal sinus (yellow circle), **(b)** with invasion of the right orbit and destruction of the medial orbital wall (yellow circle).

**Figure 2:**
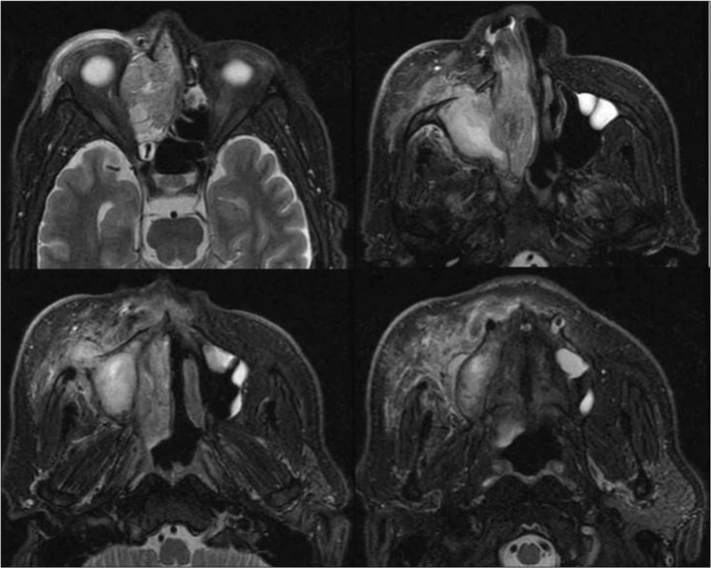
**Magnetic resonance imaging scans.** Magnetic resonance imaging reveals a tumor mass filling the right nasal cavity and infiltrating the right maxillary sinus, ethmoid sinus, and subcutaneous fat in the right buccal region.

**Figure 3:**
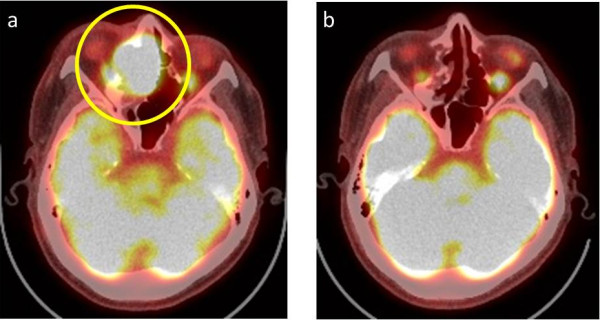
**Fluorine-18 fluorodeoxyglucose**-**positron emission tomography scans. (a)** Fluorine-18 fluorodeoxyglucose-positron emission tomography showed uptake of fluorine-18 fluorodeoxyglucose by the tumor (yellow circle). The maximum standard uptake values of the tumor obtained during fluorine-18 fluorodeoxyglucose-positron emission tomography were 14.9 at early imaging (60 minutes). **(b)** Positron emission tomography 2 years after completing proton beam therapy. There is no evidence of tumor recurrence.

**Figure 4:**
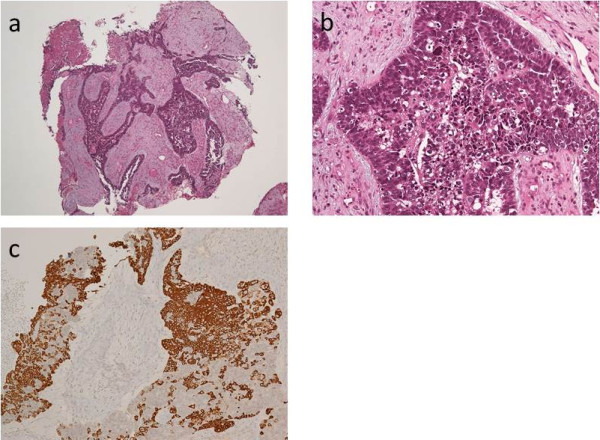
**Histological and immunohistochemical images. (a)**, **(b)** Biopsy specimen of nasal tumor shows cords of invasive basaloid cells with peripheral palisading. The tumor had hyperchromatic nuclei with a high nuclear-to-cytoplasmic ratio (hematoxylin and eosin stain). **(c)** Immunohistochemical stains of the tumor were positive for cytokeratin 14.

A 56-year-old Japanese man presented with a 1-month history of right nasal obstruction and ipsilateral slight pain in the buccal region. On examination, his right nasal cavity was filled with a hemorrhagic nasal polyp, and there was reddening and swelling of his skin in the right buccal region, and narrowing of the choroid fissure. He was admitted to our hospital. Computed tomography and magnetic resonance imaging revealed a tumor mass filling his right nasal cavity and infiltrating the right maxillary sinus, ethmoid sinus, frontal sinus, orbital floor, and orbital medial wall with erosion of bone and subcutaneous fat in the right buccal region. The tumor size was 57×52×34mm (Figures 1 and 2). Fluorine-18 fluorodeoxyglucose (FDG) positron emission tomography (PET) showed uptake of FDG by the tumor. The maximum standard uptake values of the tumor obtained during FDG-PET were 14.9 at early imaging (60 minutes; Figure 3). On histopathological examination, the tumor grew as cords with peripheral palisading and hyperchromatic nuclei with a high nuclear-to-cytoplasmic ratio. Cords of invasive basaloid cells positive for cytokeratin 14 and negative for chromogranin A and B-cell lymphoma 2 were seen (Figure 4). A diagnosis of BSCC in his right nasal cavity, stage III, T3N0M0 was made (International Union Against Cancer, 7th edition).

The risks and benefits of surgery and chemoradiotherapy were explained to the patient. He inquired about PBT. We explained the risks and benefits of PBT and its available alternatives. He decided to undergo PBT and chemotherapy. The treatment plan was decided after informed consent for PBT with concurrent chemotherapy.

The chemotherapy was single-agent, high-dose CDDP (100mg/m^2^) on days 1, 22, and 43 during PBT. He received the three courses of chemotherapy along with PBT as scheduled. Assuming a relative biological effectiveness of 1.1, the total dose of PBT was 70 cobalt Gy equivalent (CGE) in two CGE once-daily fractions, 5 days per week, for 7 weeks.

Acute and late side effects did not exceed grade 3 according to the National Cancer Institute Common Terminology Criteria for Adverse Events version 4.0. The early side effects of oral mucositis, dermatitis, and superficial keratitis of his right eye were grade 2, and hematotoxicity was grade 1. The late side effect of superficial keratitis of his right eye was grade 2, and abnormal vision in his right eye caused by retinopathy or optic nerve disorder was grade 1. FDG-PET after treatment showed no evidence of recurrence (Figure 4). He remains in complete remission (CR) 2 years after treatment.

## Discussion

BSCC in the nasal cavity and paranasal sinuses is very rare, with only 29 reported cases in the English language literature [2-6]. For advanced head and neck cancer, chemoradiotherapy is the gold standard of treatment [8,9]. In advanced BSCC, the standard of care has not been established. Our regimen used a CDDP single agent, the same as that used by Adelstein *et al.*[10]. Combination chemotherapy with CDDP and 5-fluorouracil (5-FU) is an effective regimen for head and neck cancer [8]. In 5-FU treatment, the 5-FU-related enzyme activities affect the clinical response to chemotherapy: thymidylate synthase (TS) [11]. Takemura *et al.* compared the 5-FU-related enzyme activity between esophageal BSCC and SCC. The TS activity was significantly higher in BSCC, suggesting that BSCC may have more resistance to 5-FU than SCC in the esophagus [12]. Extrapolating from their results, CDDP single-agent chemotherapy in nasal BSCC may be more effective than combination chemotherapy.

To the best of our knowledge, nasal BSCC treated with PBT has not been described in the literature. PBT has the ability to precisely localize the radiation dose, facilitating significant sparing of normal tissues in head and neck lesions [13]. The use of this feature helps mitigate the side effects of conventional radiotherapy occurring with concurrent chemotherapy.

Good local control was achieved in this case, suggesting that PBT might allow the avoidance of surgery. Given the high incidence of distant metastasis, concurrent or adjuvant chemotherapy is most probably necessary, especially for progressive lesions. In locally progressive BSCC without distant lesions, PBT concurrent with CDDP may be considered one method of treatment. It is difficult to establish high-level evidence because of the rarity of nasal BSCC, but further studies are needed to determine the optimal treatment for BSCC.

## Conclusions

We report a case of locally invasive nasal BSCC treated with PBT concurrent with CDDP. This case continues to show CR at 2-year follow-up with few side effects. Further studies are required to achieve a consensus regarding BSCC treatment, but the treatment plan adopted in our case may be one option for locally invasive BSCC.

## Consent

Written informed consent was obtained from the patient for publication of this manuscript and accompanying images. A copy of the written consent is available for review by the Editor-in-Chief of this journal.

## Abbreviations

5-FU: 5-Fluorouracil; BSCC: Basaloid squamous cell carcinoma; CDDP: Cisplatin; CGE: Cobalt Gy equivalent; CR: Complete remission; FDG: Fluorine-18 fluorodeoxyglucose; PBT: Proton beam therapy; PET: Positron emission tomography; SCC: Squamous cell carcinoma; TS: Thymidylate synthase.

## Competing interests

The authors declare that they have no competing interests.

## Authors’ contributions

All authors contributed to the care of the patient and analyzed the data. ST used all of the data available and wrote the majority of this report. KY revised the manuscript. MK, TK, and AT saw the patient in hospital and contributed the case history notes used in this report. YT contributed to image production. YK reported and provided us with the histopathological findings and slides. TK and TT were consultant radiotherapists involved in the management of this patient. All authors read and approved the final manuscript.
